# Automatic block-wise genotype-phenotype association detection based on hidden Markov model

**DOI:** 10.1186/s12859-023-05265-5

**Published:** 2023-04-07

**Authors:** Jin Du, Chaojie Wang, Lijun Wang, Shanjun Mao, Bencong Zhu, Zheng Li, Xiaodan Fan

**Affiliations:** 1 Department of Statistics, The Chinese University of Hong Kong, Shatin, New Territories Hong Kong; 2 School of Mathematical Science, Jiangsu University, Zhenjiang, Jiangsu Province China; 3 College of Finance and Statistics, Hunan University, Changsha, Hunan Province China; 4 Department of Surgery, The Chinese University of Hong Kong, Shatin, New Territories Hong Kong

**Keywords:** Hidden Markov model, Genome-Wide Association Study, Block-wise Association, EM algorithm

## Abstract

**Background:**

For detecting genotype-phenotype association from case–control single nucleotide polymorphism (SNP) data, one class of methods relies on testing each genomic variant site individually. However, this approach ignores the tendency for associated variant sites to be spatially clustered instead of uniformly distributed along the genome. Therefore, a more recent class of methods looks for blocks of influential variant sites. Unfortunately, existing such methods either assume prior knowledge of the blocks, or rely on ad hoc moving windows. A principled method is needed to automatically detect genomic variant blocks which are associated with the phenotype.

**Results:**

In this paper, we introduce an automatic block-wise Genome-Wide Association Study (GWAS) method based on Hidden Markov model. Using case–control SNP data as input, our method detects the number of blocks associated with the phenotype and the locations of the blocks. Correspondingly, the minor allele of each variate site will be classified as having negative influence, no influence or positive influence on the phenotype. We evaluated our method using both datasets simulated from our model and datasets from a block model different from ours, and compared the performance with other methods. These included both simple methods based on the Fisher’s exact test, applied site-by-site, as well as more complex methods built into the recent Zoom-Focus Algorithm. Across all simulations, our method consistently outperformed the comparisons.

**Conclusions:**

With its demonstrated better performance, we expect our algorithm for detecting influential variant sites may help find more accurate signals across a wide range of case–control GWAS.

## Background

A central problem in genetics is determining which loci of a genome are responsible for the difference between two phenotypes of an organism. A typical approach uses the case–control study, which samples subjects with both phenotypes and looks for differences in the target variables between the two groups. In the case of genotype-phenotype association study specifically, this means looking for differences between the two groups in the frequencies of alleles. A case–control Genome-Wide Association Study (GWAS) does that across a large portion of the subjects’ genomes [28], which is very helpful for elucidating disease mechanisms. For example, GWAS have identified genome loci associated with breast cancer [17, 18], ovarian cancer [21], coronary artery disease [19], type 2 diabetes [23], osteoarthritis [35] and systemic lupus erythematosus [8]. Due to the retrospective nature of case–control studies, they can prove only association rather than clear causal relationships [10] (however see [28] for follow-up methods for finding such causal relationships). Ideally, the control and case groups should be as similar as possible. An overview of GWAS experimental methodology, confounding variables that must be controlled, statistical techniques for pre- and post-processing, limitations and applications can be found in [28], while a discussion of the clinical implications of GWAS results can be found in [15]. A discussion about selecting matching controls to cases in case–control studies can be found in [3].

For GWAS, a genome can be represented as a sequence of variant sites, i.e. genomic locations that may have different genotypes. The simplest type of variant site is a single nucleotide polymorphism (SNP), in which variants differ by a single base pair. We may also simplify analysis by considering only the most common version of a gene (a major allele) and one rarer version (a minor allele). A diploid organism may have 0, 1 or 2 copies of a minor allele. In this paper, we will focus on a dichotomous phenotype *Y* and aim to detect variants which affect *Y* based on case–control SNP data.

Given case–control SNP data, the simplest GWAS methods study variant sites individually [2]. A classic model is logistic regression, where the log-odds of a subject having a phenotype is assumed to be linear in each of the variants. One may also use *p* values, for instance by performing a Fisher’s exact test at each site with the null hypothesis that the given site has no effect on the phenotype, and then making a Bonferroni correction [7]. But this approach has been criticized because Bonferroni correction is too conservative. Furthermore, it assumes that variant sites are independent, when in fact sites that influence a given phenotype tend to be physically clustered into blocks [5, 28] due to reasons such as linkage disequilibrium among others. False discovery rate (FDR) methods [7] address the first criticism by applying tougher correction to lower *p* values and a more lenient correction to larger *p* values. This improves the sensitivity; but does not address the second criticism. Uffelmann et al. [28] suggests that the Bonferroni correction factor should be the number of independent variants, rather than the total number of variants; but this requires knowledge of which sites are independent. Uffelmann et al. [28] also suggests that the baseline threshold of 0.05 may need to be adjusted depending on intended population size and minimum detectable minor allele frequency (MAF).

More recent algorithms for identifying influential variant sites take into account the spatial clustering [2]. For instance, one approach is to group variant sites into blocks by linkage disequilibrium, and then recognize only associations that are confirmed by other variant sites in the same block [24]. Another approach is to collapse blocks of variant sites, where a block is counted as On if any site in the block has a minor allele and Off if none of them do [11]. Those two assume prior knowledge of the blocks. A more flexible approach is found in [31]: the Zoom-Focus algorithm (ZFA). In the zoom step, the algorithm divides the genome into a binary tree structure and tests the significance of each half. In the focus step, the algorithm enumerates all possible adjustments of the boundaries of the halves, and tests their significance. Testing relies on other existing algorithms to obtain *p* values. The paper uses 4 such: SKAT [32], SKAT-O [9], burden [25] and wtest [27]. In this paper, we use as comparison the Fisher’s exact test with Bonferroni correction, the Benjamini–Hochberg FDR method, and ZFA with each of its 4 testing methods.

Yet another class of methods uses a deep-learning approach. For example, [14] applies convolutional neural networks to GWAS data (note that they assume a continuous rather than dichotomous phenotype). Their method has the further advantage of bypassing the need to impute missing genotypes. The first focus of their method is to train the network to predict phenotype given a test genotype. The influence strength of individual SNPs is then measured using saliency values. These are calculated for each subject by taking the maximum gradient with respect to variables encoding the genotype at the given site. Then the overall influence strength is measured by taking the median saliency value over the testing set. In general, convolutional neural networks can take into account clustering by grouping nearby sites into the same kernel window, information about which will then be summarized and passed to the next layer. But a limitation is that it may not be clear which kernel window size to use in a given application. Complicating that further, there may be multiple influential blocks of different lengths, which would suggest the need for multiple kernel window sizes. Yet another issue with machine learning methods in general is that they require training data, i.e. a pre-existing set of sites with known influential/not-influential state.

We propose modelling the unknown genotype-phenotype association state sequence using a Hidden Markov model (HMM). HMM offers a different outlook on association state clustering to existing methods. Unlike some methods such as collapsing, it does not require knowing the locations of the blocks ahead of time. Furthermore, it attempts to offer more than ZFA by finding not just locations of blocks, but a model for their formation: Blocks form because the state of each variant site affects the state of the next site. Also, our model is designed to accommodate both rare and common variants, making it more versatile than methods that perform well on only rare or only common variants. HMM has already been applied successfully in a wide variety of settings including speech recognition [22], image classification [12], musical key detection [20], precipitation [36], evolution [4] and gene segmenting [6]. Some literature studied the validity of the Markov property for DNA sequences [26, 29, 34]. Most relevantly for us, HMM has been applied to the identification of genes encoding a particular phenotype in [16]; there, the phenotype is a variant surface protein in a particular disease-causing parasite.

We will regard variant sites as being in one of three possible unobserved states: Negative Influence, No Influence and Positive Influence. The influence of minor alleles on the phenotype will depend on which state the variant site is in. The states themselves will be governed by a Markov process, with the state of each variant site affecting the state of the next according to a transition probability matrix. The goal of the algorithm will be to determine the state of each variant site as accurately as possible. After describing the algorithm in detail, we will compare its performance to the Fisher’s exact test with Bonferroni correction, FDR and ZFA.

## Methods

Suppose we have collected the genotypes and phenotypes of *n* subjects, with each genotype consisting of *p* variant sites. Let *N* and *M* be the numbers of phenotype 0 (control) and phenotype 1 (case) subjects respectively. We assume that each site has one possible minor allele. The data will be organized in the form of a *genotype matrix* *G* of size $n\times p$, with the (*i*, *j*)th entry representing the number of minor allele copies of the *j*th variant that the *i*th subject has, and a *phenotype vector* *y* of length *n*, whose *i*th component is the phenotype of the *i*th subject.

We assume that there are 3 possible states for a variant site: Negative Influence, No Influence and Positive Influence. We write $S_j=-1,0,1$ for the *j*th variant site being in each of these states respectively. The goal of HMM is to predict a state sequence $\langle s_j:j=1,\ldots ,p\rangle$ that best fits the observed data. HMM will have the following parameters:

- A null distribution $[p_0^j,p_1^j,p_2^j]$ for each variant site, representing the probability of a phenotype 0 subject having 0, 1 and 2 copies of the minor allele at the *j*th site.
- Influence strength parameters $\theta ^-_0,\theta ^-_1,\theta ^+_0,\theta ^+_1$, controlling the magnitude by which the distribution for phenotype 1 subjects departs from that of phenotype 0 subjects. The superscript indicates whether the parameter affects Negative Influence or Positive Influence. The subscript indicates whether the direct intent is to shift probability mass towards/away from genotype ‘AA’ or ‘aa’. See the “Appendix” for more details.
- Markov parameters $\pi$ and *A*, where $\pi =(\pi _{-1},\pi _0,\pi _1)$ is the vector of probabilities of the first variant site being in states $-1,0,1$ and $A=(a_{k\ell })_{k,\ell =-1,0,1}$ is the matrix of probabilities of transitioning to state $\ell$ in the next site given that the current site is in state *k*.

For brevity, we write $\vec{p}$ for $\{[p_0^j,p_1^j,p_2^j]:j=1,\ldots ,p\}$, $\theta$ for $\{\theta ^-_0,\theta ^-_1,\theta ^+_0,\theta ^+_1\}$ and $\tau =\{ \vec{p} ,\theta ,\pi ,A\}$ for the set of all parameters. At each variant site *j* and for each state $k=-1,0,1$, HMM will produce parameterized emission distributions $f_k^j(x| \vec{p} ,\theta )$, that give the probability of observing contingency table *x* at that site.

In additional to the model parameters described above, our HMM uses thresholding parameters to guarantee biologically meaningful and practically identifiable states. The minimum influence strength threshold $\theta _{min}$ sets a lower bound on allowable values for $\theta _0^-,\theta _0^+,\theta _1^-,\theta _1^+$. This threshold is necessary because very low values of $\theta _0^-$ and $\theta _1^-$ (respectively $\theta _0^+$ and $\theta _1^+$) make the Negative Influence (respectively Positive Influence) state almost indistinguishable from No Influence. This leads to the collapse of either sensitivity or specificity. We cautiously recommend $\theta _{min}=0.15$, but with caveats, for reasons that will be explained in later sections. The self-transition threshold $a_{min}$ sets a lower bound for the diagonal entries of the Markov transition matrix *A*. It is necessary to ensure that predicted influential sites come in blocks, with higher threshold values leading to longer predicted blocks. We used and recommend $a_{min}=0.5$.

HMM attempts to find the values of $\vec{p} ,\theta ,\pi ,A$ that maximize the probability of obtaining the observed data. The master objective function is the logarithm of that probability. The algorithm is as follows:

1. Make starting estimates for $\vec{p} ,\theta ,\pi ,A$
2. Evaluate the master objective function
3. Using the values for $\vec{p} ,\theta ,\pi ,A$, evaluate the emission distribution functions at the observed values. Then calculate the forward and backward variables, update the marginal state probabilities, and perform the Viterbi Algorithm to update the most probable state sequence.
4. Using the marginal state probabilities, update the parameters values with the Expectation-Maximization Algorithm.
5. Enforce thresholds on self-transition probabilities $A_{kk}$, $k=-1,0,1$ and $\theta$.
6. Repeat steps 2–5 until the master objective function has increased by less than a threshold $\epsilon$. Then output the most probable state sequence from the iteration with highest master objective value and halt.

The forward $\alpha$ and backward $\beta$ variables are explained in [30]. $\alpha _j(k)$ is the probability of obtaining the observed sequence of contingency tables up to including the *j*th site and being in state *k* at the *j*th site. $\beta _j(k)$ is the probability of obtaining the observed sequence of contingency tables after but not including the *j*th site and given that the state is *k* at the *j*th site. The master objective function is calculated as $\alpha _p(-1)+\alpha _p(0)+\alpha _p(1)$. Marginal state probabilities are calculated as $\gamma _j(k)=\frac{\alpha _j(k)\beta _j(k)}{\alpha _j(-1)\beta _j(-1)+\alpha _j(0)\beta _j(0)+\alpha _j(1)\beta _j(1)}$, where $\beta$ is the backward variable. We use $\epsilon =0.01$.

**Fig. 1:**
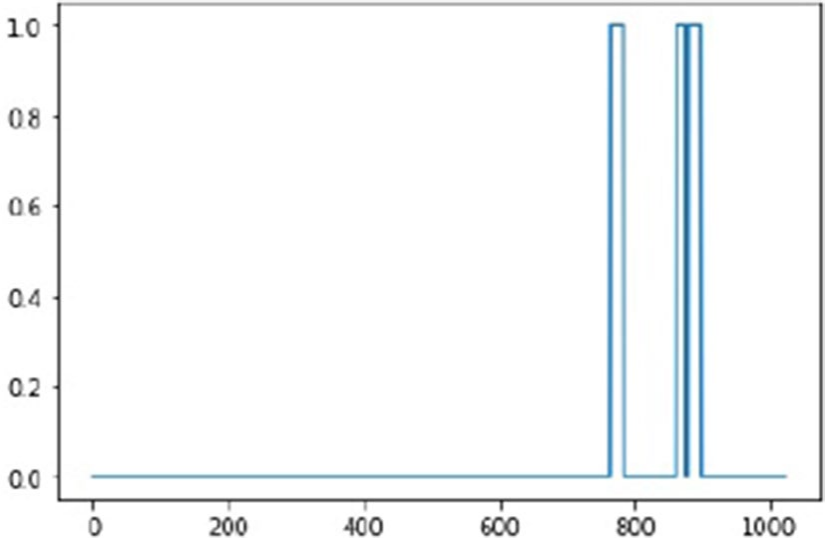
True state of each variant site. 1 means negative influence or positive influence, 0 means no influence

**Fig. 2:**
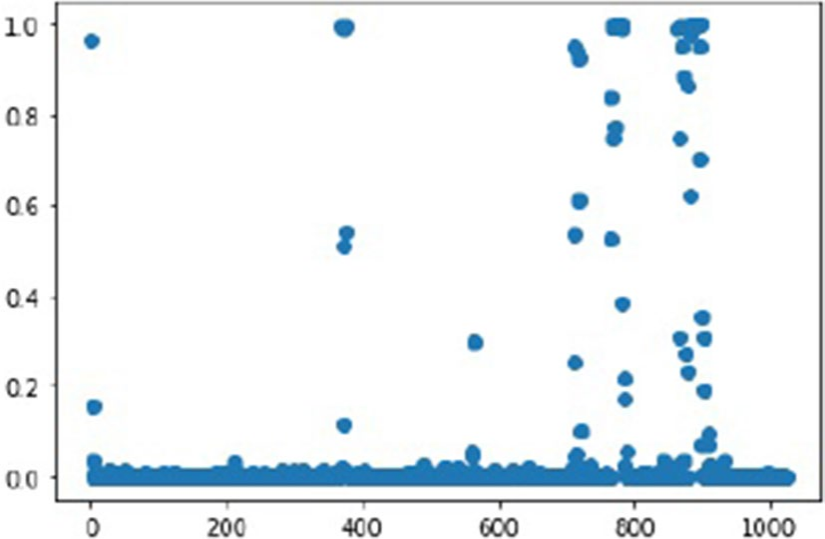
Marginal probability of being in negative influence or positive influence state, as predicted by HMM

We may also report the marginal state probabilities $\gamma _j(k)$ from the iteration with highest master objective value. These do not necessarily capture the most probable state sequence; but are useful if we want to calibrate a balance between sensitivity and specificity, for instance when interpolating a ROC (receiver operating characteristic) curve. The entire algorithm is summarized in a flowchart in the “Appendix”. Example plots of the marginal state probabilities, and the true states of the variant sites, from one run (HMM threshold 0.1, Default Initials, trial 1, Simulation Group 2) is shown in Figs. 1 and 2. To simplify the plot, Negative Influence and Positive Influence are collapsed into one, as are their corresponding marginal probabilities.

We will describe in detail the emission distribution functions, EM parameter re-estimation process and initial parameter estimates. We omit an explanation of Step 3 because that is already explained in detail in [30]. As one last consideration before we begin, note that many probabilities we work with will grow or shrink exponentially with number of subjects or number of variant sites, which quickly leads to overflow or underflow errors. Viterbi [30] suggests using a scaling constant to keep numbers within machine limits. We found it was simpler to store only the logarithms of the probabilities. Thus while the formulas in this paper are shown in their original form, their implementations in computer code are in terms of the logarithms of the probability variables.

Evaluation of our method, including descriptions of the simulations used to do so, are in the “Results” section.

### Emission distribution functions

Classical HMM assumes we have an observation sequence [22]. For that we use the sequence of $2\times 3$ contingency tables for each variant site. Suppose the *j*th contingency table $x_j$ is:

**Table:** 

$n_0$	$n_1$	$n_2$
$m_0$	$m_1$	$m_2$

where $n_i$ is the number of phenotype 0 subjects with *i* copies of the minor allele and $m_i$ is the number of phenotype 1 subjects with *i* copies of the minor allele.

We assume that among the population of phenotype 0 individuals, there are underlying probabilities $p_0,p_1,p_2$ for having 0, 1, 2 copies of the minor allele respectively. Similarly, let $q_0,q_1,q_2$ be the same for the population of phenotype 1 individuals. Then the probability of obtaining the contingency table above, and hence the emission distribution function, is given by $\frac{N!}{n_0!n_1!n_2!}p_0^{n_0}p_1^{n_1}p_2^{n_2}\frac{M!}{m_0!m_1!m_2!}q_0^{m_0}q_1^{m_1}q_2^{m_2}$. The $p_i$ are fixed by the null distribution parameters while the $q_i$ depend on the state of the variant site.

In the No Influence state, $q_i=p_i$. In the Negative Influence state, $(q_0,q_1,q_2)=(\frac{e^{\theta ^-_0}p_0}{e^{\theta ^-_0}p_0+p_1+e^{-\theta ^-_1}p_2},\frac{p_1}{e^{\theta ^-_0}p_0+p_1+e^{-\theta ^-_1}p_2},\frac{e^{-\theta ^-_1}p_2}{e^{\theta ^-_0}p_0+p_1+e^{-\theta ^-_1}p_2})$. In the Positive Influence state, $(q_0,q_1,q_2)=(\frac{e^{-\theta ^+_0}p_0}{e^{-\theta ^+_0}p_0+p_1+e^{\theta ^+_1}p_2},\frac{p_1}{e^{-\theta ^+_0}p_0+p_1+e^{\theta ^+_1}p_2},\frac{e^{\theta ^+_1}p_2}{e^{-\theta ^+_0}p_0+p_1+e^{\theta ^+_1}p_2})$. A more detailed explanation of these formulas can be found in the “Appendix”.

### The EM step

Let $\tau ^{(t)}$ denote the value of $\tau$ after *t* iterations, i.e. *t* passes through steps 2–5 in the overall algorithm, and similarly for any other parameter or set of parameters. The purpose of the Expectation-Maximization step is to update the parameter values: Given $\tau ^{(t)}$, find $\tau ^{(t+1)}$ that better fits the observations. EM consists of two steps.

In the E step, we find the function to be maximized. First, we find HMM’s likelihood function: $L(x,S|\tau )=\prod _{j=1}^p\prod _{s\in \{-1,0,1\}}(f_s^j(x_j| \vec{p} ,\theta )P(S_j=s|\pi ,A,S_{j-1}))^{1_{S_j=s}}$.

The function to be maximized is:

$$E_{S|\tau ^{(t)},x}[\log L(x,S|\tau ])]$$

$$=\sum _{j=1}^p\sum _{s\in \{-1,0,1\}}\gamma _j^{(t)}(s)(\log (f_s^j(x_j| \vec{p} ,\theta ))+\log P(S_j=s|\pi ,A,S_{j-1}))$$,

where $\gamma _j^{(t)}(s)=P(S_j=s|x,\tau ^{(t)})$ was calculated in Step 3.

In the M step, we find values of $\vec{p} ,\theta ,\pi ,A$ that maximize this function and take $\tau ^{(t+1)}$ to be this new set of values. Details are in the “Appendix”.

### Initial parameter estimates

The last main component of HMM we will discuss is the choice of initial estimates for the model parameters. The EM algorithm is designed to converge to a local maximum, not necessarily the global maximum; hence HMM may likewise converge to a local but non-global maximum. In this subsection, we provide two choices of initial estimates called Default and Random. Default will always yield the same output, and hence need only be run once, while Random may produce a different output each time, and hence may be run multiple runs. The run with the highest final master objective value should be selected.

#### Default initials

At variant site *j*, recall that $p_i^j$ is the underlying probability that a phenotype 0 subject has *i* copies of the minor allele. Hence a reasonable initial estimate is $\frac{n_i}{N}$. To avoid numerical errors, we replace $n_i$ with the pseudocount 0.5 in case it is 0. This forms our estimate for $\vec{p} ^{(0)}$.

In the “Appendix”, we see that:

$$\begin{aligned}(\theta _0^-)^{(t+1)},(\theta _1^-)^{(t+1)}=\mathop {\mathrm {arg\,max}}\limits \sum _{j=1}^p\gamma _j^{(t)}(-1)\log (f_{-1}^j(x_j| \vec{p} ,\theta _0^-,\theta _1^-)),\\(\theta _0^+)^{(t+1)},(\theta _1^+)^{(t+1)}=\mathop {\mathrm {arg\,max}}\limits \sum _{j=1}^p\gamma _j^{(t)}(1)\log (f_1^j(x_j| \vec{p} ,\theta _0^+,\theta _1^+)).\end{aligned}$$

Motivated by this, we define the strengths of the *j*th variant site as follows:

$$\begin{aligned}\theta _0^-(j),\theta _1^-(j)=\mathop {\mathrm {arg\,max}}\limits [\log (f_{-1}^j(x_j| \vec{p} ,\theta _0^-,\theta _1^-))],\\\theta _0^+(j),\theta _1^+(j)=\mathop {\mathrm {arg\,max}}\limits [\log (f_1^j(x_j| \vec{p} ,\theta _0^+,\theta _1^+))].\end{aligned}$$

We search for these maxima using the alternating, one variable at a time, method in the “Appendix”. Once the strengths of all variant sites have been calculated, we use as our initial estimate: $\theta _0^-=\text {median}\{\theta _0^-(j):\theta _0^-(j)>\theta _{min}\}$, unless this set is empty, in which case we take $\theta _0^-=\theta _{min}$. Initial estimates for the other three components of $\theta$ are defined analogously.

Next, we make initial estimates of $P(S_j=k|x, \vec{p} ,\theta )$. We take $\frac{f_k^j(x_j| \vec{p} ,\theta )}{\sum _{k=-1}^1f_k^j(x_j| \vec{p} ,\theta )}$, the Bayes’ posterior distribution given a prior distribution of $[\frac{1}{3},\frac{1}{3},\frac{1}{3}]$. To avoid numerical errors from probabilities too close to 0 or 1, we set a threshold minimum probability of $e^{-30}$, and then rescale so that the three probabilities sum to 1. Call these quantities $b_j^k$.

We then use as initial estimates: $\pi _k=b_1^k$ and $a_{k,\ell }=\frac{\sum _{j=1}^{p-1}b_j^k b_{j+1}^{\ell }}{\sum _{j=1}^{p-1}b_j^k}$, where $a_{k,\ell }$ is the entry in the *k*th row and $\ell$th column of *A*. The latter expression is the expected number of transitions from *k* to $\ell$ divided by the expected number of occurrences of *k* before the final site, if the sites were independent.

#### Random initials

Alternatively, we can start the EM algorithm with random parameter values. To explain the random initial, we first define a random triple function: sample $u_0,u_1,u_2$ independent and identically distributed (i.i.d.) from *U*(0, 1), sort the set $\{\frac{u_0}{u_0+u_1+u_2},\frac{u_1}{u_0+u_1+u_2},\frac{u_2}{u_0+u_1+u_2}\}$ from largest to smallest, then output the set.

$[p_0^j,p_1^j,p_2^j]$ are chosen i.i.d. for each variant site by calling the random triple function. $\theta ^-_0,\theta ^-_1,\theta ^+_0,\theta ^+_1$ are chosen by sampling i.i.d. from *U*(0, 1). $\pi =[\pi _{-1},\pi _0,\pi _1]$ is chosen by calling the random triple function, except that while $\pi _0$ is taken to be the largest of the values, which of the two remaining values should be taken as $\pi _{-1}$ and which as $\pi _1$ is decided by a 50–50 Bernoulli draw. $A=(a_{k,\ell })_{k,\ell =-1,0,1}$ is chosen as follows: The diagonal entries are drawn from *U*(0.5, 1). Then for each row, the remaining probability is distributed in proportion $t,1-t$ between the remaining two entries, where *t* is drawn from *U*(0, 1).

## Results

We carried out nine groups of simulations, each consisting of 20 trials, except for groups 7 and 8 which consisted of 40 trials each. Each trial consisted of running HMM together with 6 comparison methods on a synthesized dataset where the true state of each variant site is known. We recorded the sensitivity, specificity and MCC (Matthews correlation coefficient) of each method in each trial and calculated means. In trials with no true influential sites, sensitivity and MCC were marked as ‘NA’ and excluded from the mean. Unless otherwise stated, the trials consisted of 1000 phenotype 0 subjects and 1000 phenotype 1 subjects. Although HMM results distinguish between Negative Influence and Positive Influence, we collapsed these two categories into one for the purposes of calculating sensitivity and MCC averages. A table displaying the means is shown near the end of each of the following subsections. Standard deviations of MCCs are shown in parentheses.

HMM was run with three $\theta$ thresholds: 0.1, 0.15 and 0.2. Within each threshold value, HMM was run 4 times: Once with Default initials and three times with Random initials, and only the output of the run with the highest final value of the master objective function was recorded as HMM’s “official” output. For comparison purposes, HMM with each of the three thresholds was run and reported as if it were a separate method.

The comparison methods used were: Bonferroni, FDR and ZFA with four *p* value methods: SKAT, SKATO, burden and wtest. The first two consisted of extracting a *p* value from the Fisher’s exact test separately for each variant site. Bonferroni marked the site as influential if the *p* value was below 0.05 divided by the number of variant sites. We use a version of FDR due to Benjamini and Hochberg [1], which ranks the variant sites by *p* value (the site with lowest *p* value having rank 1, the site with second lowest having rank 2, etc.) and multiplies Bonferroni’s significance threshold by the *p* value ranking. The four ZFA methods were run from the package zfa in R; we used CommonRare_Cutoff = 0.5, fast.path = FALSE and called each of the four methods with the test argument.

The simulation groups we used are summarized in Table 1. Sample Size is the number of phenotype 0 subjects, followed by the number of phenotype 1 subjects per dataset. Site Strengths is the way Negative Influence and Positive Influence sites’ degree of influence were set: ‘HMM’, which has its own influence strength parameters, ‘MAF-dependent’ (see “First block model simulations” section) or ‘uniform’ (see any of the later subsections). MAFs is the way minor allele frequencies were generated: ‘mixed’ (see “First block model simulations” section) or ‘low’ (see “Low MAFs” section). Noise Terms is whether or not two additional terms, unrelated to genotype, were included in the phenotype logit-probability equation (see third subsection). Impurity is the probability that a site in an influential block is No Influence.

**Table 1:** Summary of simulation groups

Group	Model	Sample size	Site strengths	MAFs	Noise terms	Impurity
1	HMM	1000,1000	HMM	–	–	–
2	Block	1000,1000	MAF-dependent	Mixed	No	0
3	Block	1000,1000	Uniform	Mixed	Yes	0.25
4	Block	1000,1000	Uniform	Mixed	Yes	0.5
5	Block	1500,500	Uniform	Mixed	No	0
6	Block	1500,500	Uniform	Mixed	Yes	0.25
7	Block	150,150	Uniform	Mixed	No	0
8	Block	200,100	Uniform	Mixed	No	0
9	Block	2500,2500	Uniform	Low	No	0

### HMM model simulations

As a preliminary test for our method, the first group of simulations generated data according to the HMM model itself. High performance for HMM on these datasets was thus expected. First, we generated values for $\vec{p} ,\theta ,\pi ,A$ similarly to as in Random Initials but with some differences:

- $\vec{p}$ was generated the same as in Random Initials.
- Each component of $\theta$ was sampled from *U*(0.05, 1). The lower bound prevented influences from being too weak.
- $\pi$ was generated the same as in Random Initials.
- The diagonal entries were $a_{0,0}=0.5+0.5\beta _1$, $a_{-1,-1}=0.5+0.5\beta _2$ and $a_{1,1}=0.5+0.5\beta _3$, where $\beta _1$ was drawn from $\text {Beta}(99,1)$ and $\beta _2,\beta _3$ were drawn from $\text {Beta}(90,10)$. For the off-diagonal entries, draw $t_1$ from *U*(0, 1) and $t_2,t_3$ from $\text {Beta}(99,1)$. Then $a_{0,-1}=t_1(1-a_{0,0})$, $a_{0,1}=(1-t_1)(1-a_{0,0})$, $a_{-1,0}=t_2(1-a_{-1,-1})$, $a_{-1,1}=(1-t_2)(1-a_{-1,-1})$, $a_{1,0}=t_3(1-a_{1,1})$ and $a_{1,-1}=(1-t_3)(1-a_{1,1})$.

We then generated a true state sequence $\langle \hat{s}_j:j=1,\ldots ,1024\rangle$, with $\pi$ used to generate $\hat{s}_1$ and *A* used to generate $\hat{s}_{j+1}$ from $\hat{s}_j$.

At each variant site *j*, using the true state $\hat{s}_j$ as well as $\theta$ and the null distribution $[p_0^j,p_1^j,p_2^j]$, we calculated the phenotype 1 distribution $[q_0^j,q_1^j,q_2^j]$ from the formulas in Emission Distribution Functions. Then we generated the contingency tables by sampling from the multinomial distributions: $\text {multinomial}(1000,[p_0^j,p_1^j,p_2^j])$ and $\text {multinomial}(1000,[q_0^j,q_1^j,q_2^j])$.

**Fig. 3:**
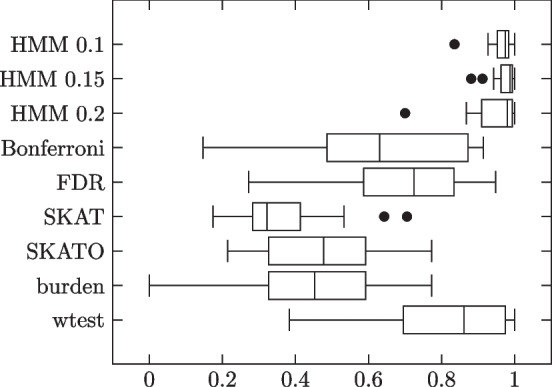
Individual trial MCCs

Lastly, we generated the genotypes and phenotypes as follows: The first 1000 subjects were phenotype 0 while the second 1000 were phenotype 1. Thus the phenotype vector consisted of 1000 0’s followed by 1000 1’s. As for the genotype matrix, we knew that the first 1000 rows would correspond to phenotype 0 subjects while the second 1000 rows would correspond to phenotype 1 subjects. We furthermore knew from the contingency tables, for each variant site, how many subjects of phenotype 0 had 0 copies of the minor allele, how many had 1, how many had 2, and the same for phenotype 1 subjects. To determine which phenotype 0 subjects had each genotype, we randomly shuffled a temporary copy of the list of subjects and filled the quota for genotype 0, followed by genotype 1, followed by genotype 2, according to the shuffled order. Then we did the same for phenotype 1 subjects. This process was repeated for each variant site. Results summarized in Table 2 and Fig. 3.

**Table 2:** HMM model simulation results. 1000 phenotype 0, 1000 phenotype 1 subjects

Method	Sensitivity	Specificity	MCC
HMM 0.1	0.9945	0.9950	0.9598 (0.0373)
HMM 0.15	0.9849	0.9981	0.9712 (0.0327)
HMM 0.2	0.9420	0.9977	0.9409 (0.0764)
Bonferroni	0.4679	0.9999	0.6215 (0.2432)
FDR	0.8040	0.9748	0.6877 (0.1875)
SKAT	0.2009	0.9941	0.3676 (0.1458)
SKATO	0.3099	0.9861	0.4610 (0.1715)
burden	0.3023	0.9872	0.4493 (0.1928)
wtest	0.8688	0.9710	0.8163 (0.1850)

Each dataset was generated according to HMM, with the parameters generated as above

As expected, HMM outperforms all comparison methods. HMM does well under all three $\theta$ thresholds, but does best with 0.15.

### First block model simulations

The rest of the simulations generated data according to a block model, intended to be neutral between HMM and the comparison methods. Influential sites were assumed to come in blocks of defined length and position. Unlike in the HMM model, genotypes were fixed before phenotypes, with the former assumed to exert a causal effect on the latter. We modeled this causal effect with a logistic regression equation from Wu et al. [32]: $\text {logit}P(Y=1)=\beta +\sum _{j=1}^p\beta _jG_j$, where $\beta _j$ is a coefficient measuring the effect of the *j*th variant and $\beta$ is a constant term. Larger values of $\beta _j$, either positive or negative, imply the variant is more influential.

Prior to starting any of the individual synthesized datasets, we first fixed a distribution for number of influential sites as follows: We generated 10,000 datasets as in HMM Model Simulations, but saved only the number of influential sites, and excluded instances with more than 300 influential sites. Then, for each block model simulation, we sampled one number randomly from this list, and took this as our approximate number of influential sites.

Next, we decided the influential blocks. For each simulation, we set the number of blocks as 1, 2, 3 or 4 with equal probability. Having decided this, for each block the length was sampled i.i.d. from $Poisson(\frac{\#(\text {influential sites})}{\#(\text {blocks})})$, with length 0 replaced by 1 if this sample drew a 0. The locations of the blocks were selected randomly with uniform probability, subject only to the constraint that blocks could not overlap. Then each block was set to either Negative Influence or Positive Influence with 50–50 probability, while sites outside of any block were marked No Influence. This formed the true state sequence.

Then we generated the genotypes and phenotypes. For each variant site *j*, the minor allele frequency *q* was $q=0.001\cdot 500^u$, where $u\sim U(0,1)$ i.i.d. That is, MAFs could range from 0.1 to $50\%$ with more weight on lower MAFs. Following [31, 32] we then set $\beta _j=\pm 0.3|\log _{10}(q)|$ so that rarer minor alleles had stronger influence. The full genotype of a prospective subject was set by choosing the number of minor alleles independently for each variant site according to the Hardy Weinberg Formula for each site’s value of *q*. Recall that in this group of simulations, $\text {logit}P(Y=1)=\beta +\sum _{j<p}\beta _jG_j$, where the constant term $\beta$ controls the proportion of phenotype 0 subjects to phenotype 1 subjects. In order to produce an expected number of phenotype 0 subjects equal to the desired number, $\beta$ should be set to $-\mu +log(b/a)$, where *a* is the desired number of phenotype 0 subjects, *b* is the desired number of phenotype 1 subjects and $\mu =E(\sum _{j=1}^p\beta _jG_j)$. To estimate $\mu$, 1000 genotypes were generated as above, and $\mu$ was taken to be the mean. We then filled in the desired number of subjects for our simulation using a quota system: We generated a genotype $G_i$ as above, followed by a phenotype $y_i$ set to 1 with logit probability as above, and 0 otherwise. The subject was then added to the study if and only if the relevant phenotype’s quota had not yet been filled; and this process was repeated until both quotas were filled. As a final step for the benefit of the ZFA methods, variant sites where no subjects had at least one copy of the minor allele were removed.

**Fig. 4:**
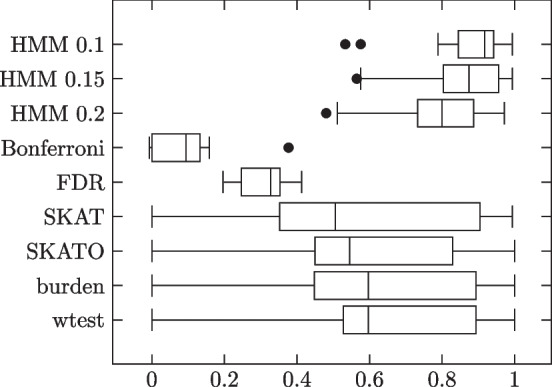
Individual trial MCCs

These simulations are conceptually more challenging than the HMM model simulations because whereas in the HMM model each influential site directly affects the corresponding column of the genotype matrix, in the block model, all such influences are mixed together under a sum, which is then further hidden behind a dichotomous variable. Thus, even before considering the specifics of a classification algorithm, lower classification performance is to be expected. Results summarized in Table 3 and Fig. 4.

**Table 3:** Block model simulation results: 1000 phenotype 0, 1000 phenotype 1 subjects

Method	Sensitivity	Specificity	MCC
HMM 0.1	0.8543	0.9926	0.8700 (0.1282)
HMM 0.15	0.7936	0.9958	0.8469 (0.1327)
HMM 0.2	0.6739	0.9965	0.7719 (0.1427)
Bonferroni	0.0173	0.9999	0.0886 (0.0927)
FDR	0.2546	0.9816	0.3074 (0.0610)
SKAT	0.7456	0.9019	0.5649 (0.3174)
SKATO	0.6672	0.9383	0.5754 (0.2924)
Burden	0.6090	0.9572	0.6111 (0.3048)
wtest	0.6034	0.9609	0.6356 (0.2558)

Influential site strengths are set higher for rarer minor alleles. No added noise terms. Pure blocks

HMM again outperforms all comparison methods. HMM does best with threshold 0.1; performance declines with increasing threshold value.

### Block model with i.i.d. site strengths, noise terms and impurity

We generated two more groups of block model simulations, but with the following modifications:

- $\beta _j\sim \pm U(0.6,1.2)$. This was the range of possible individual site strengths in the simulations from [31], but with the dependence on MAF removed and replaced with an i.i.d. uniform draw.
- Noise Terms. Following [31], we add two noise terms to the logistic regression equation: $0.5X_1+0.5X_2$, where $X_1$ is a standard normal variable and $X_2$ is a 50–50 coin flip between 0 and 1.
- Impurity: Sites within an influential block had a probability of reverting to No Influence.

**Fig. 5:**
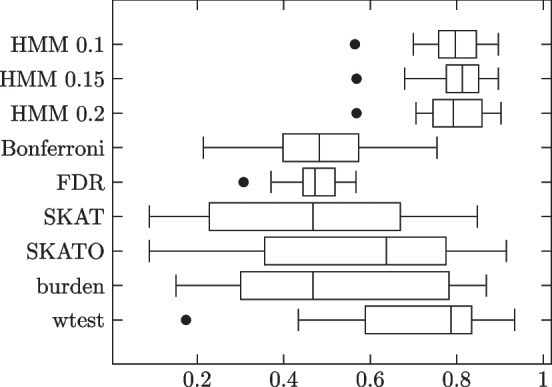
Individual trial MCCs

**Fig. 6:**
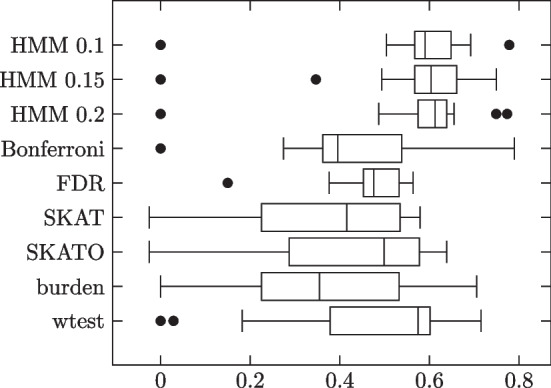
Individual trial MCCs

In the first additional simulation group, impurity was set to 0.25. In the second, it was set to 0.5. To compensate, the approximate number influential sites was scaled by 4/3 and 2 respectively. Results summarized in Tables 4, 5 and Figs. 5, 6.

**Table 4:** Block model simulation results: 1000 phenotype 0, 1000 phenotype 1 subjects

Method	Sensitivity	Specificity	MCC
HMM 0.1	0.8533	0.9721	0.7908 (0.0815)
HMM 0.15	0.8428	0.9774	0.7963 (0.0807)
HMM 0.2	0.8243	0.9816	0.7936 (0.0803)
Bonferroni	0.2631	1.0000	0.4843 (0.1304)
FDR	0.4741	0.9805	0.4734 (0.0654)
SKAT	0.5297	0.9266	0.4509 (0.2406)
SKATO	0.6622	0.9371	0.5651 (0.2464)
Burden	0.5232	0.9533	0.5091 (0.2494)
wtest	0.7218	0.9657	0.7024 (0.1890)

Influential site strengths independent of MAFs. Added noise terms. Impurity 0.25

**Table 5:** Block model simulation results: 1000 phenotype 0, 1000 phenotype 1 subjects

Method	Sensitivity	Specificity	MCC
HMM 0.1	0.6028	0.9685	0.5731 (0.1576)
HMM 0.15	0.6156	0.9724	0.5670 (0.1653)
HMM 0.2	0.5805	0.9782	0.5774 (0.1611)
Bonferroni	0.2379	1.0000	0.4393 (0.1813)
FDR	0.4626	0.9817	0.4661 (0.0934)
SKAT	0.5088	0.9126	0.3742 (0.1767)
SKATO	0.5840	0.9115	0.4171 (0.1920)
Burden	0.4052	0.9330	0.3476 (0.1946)
wtest	0.6396	0.9184	0.4719 (0.2105)

Influential site strengths independent of MAFs. Added noise terms. Pure blocks

HMM outperforms the comparison methods under both impurity levels. Different thresholds don’t change the performance very much. All methods tend to perform worse with the higher impurity level.

### Block model with imbalanced phenotypes

**Fig. 7:**
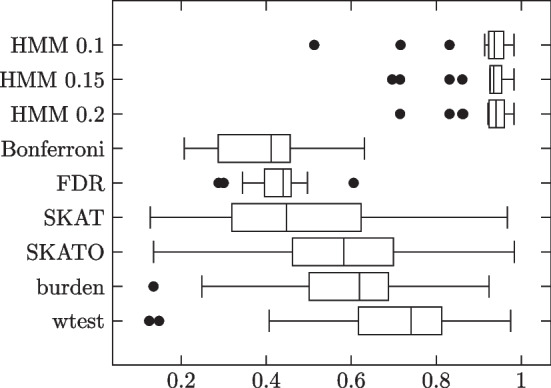
Individual trial MCCs

**Fig. 8:**
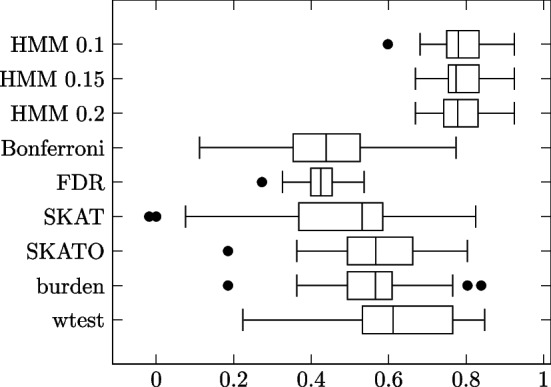
Individual trial MCCs

Next, we generated two groups of block model simulations with imbalanced phenotypes, i.e. more phenotype 0 subjects than phenotype 1. Specifically, we used 1500 of the former and 500 of the latter. The first group featured just the imbalanced phenotypes and the uniform i.i.d. individual site strengths, with the simulations otherwise set up as in the first group of block model simulations. The second group added to this the noise terms and an impurity of 0.25 as in the preceding subsection. Results summarized in Tables 6, 7 and Figs. 7, 8.

**Table 6:** Block model simulation results: 1500 phenotype 0, 500 phenotype 1 subjects

Method	Sensitivity	Specificity	MCC
HMM 0.1	0.8960	0.9949	0.9018 (0.1115)
HMM 0.15	0.9087	0.9943	0.9082 (0.0797)
HMM 0.2	0.9135	0.9949	0.9211 (0.0640)
Bonferroni	0.1861	1.000	0.3980 (0.1320)
FDR	0.3867	0.9798	0.4292 (0.0717)
SKAT	0.5342	0.9380	0.4820 (0.2129)
SKATO	0.5789	0.9499	0.5852 (0.2017)
Burden	0.4931	0.9712	0.5866 (0.1822)
wtest	0.6692	0.9597	0.6812 (0.2358)

Influential site strengths independent of MAFs. No added noise terms. Pure blocks

**Table 7:** Block model simulation results: 1500 phenotype 0, 500 phenotype 1 subjects

Method	Sensitivity	Specificity	MCC
HMM 0.1	0.8620	0.9717	0.7786 (0.0707)
HMM 0.15	0.8611	0.9718	0.7828 (0.0626)
HMM 0.2	0.8230	0.9750	0.7845 (0.0597)
Bonferroni	0.2208	1.000	0.4302 (0.1620)
FDR	0.4060	0.9797	0.4187 (0.0623)
SKAT	0.6059	0.9121	0.4611 (0.2418)
SKATO	0.6834	0.9178	0.5671 (0.1495)
Burden	0.6417	0.9251	0.5602 (0.1505)
wtest	0.7781	0.9161	0.6124 (0.1762)

Influential site strengths independent of MAFs. Added noise terms. Impurity 0.25

HMM outperforms the comparison methods in both groups. All three threshold values show similar performance. All methods show worse performance in the noise and impurity group, except Bonferroni.

### Block model with small sample size

**Fig. 9:**
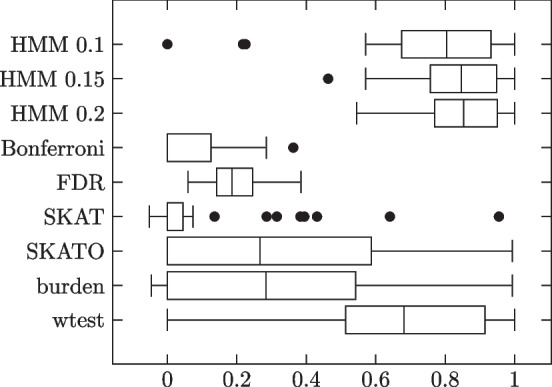
Individual trial MCCs

**Fig. 10:**
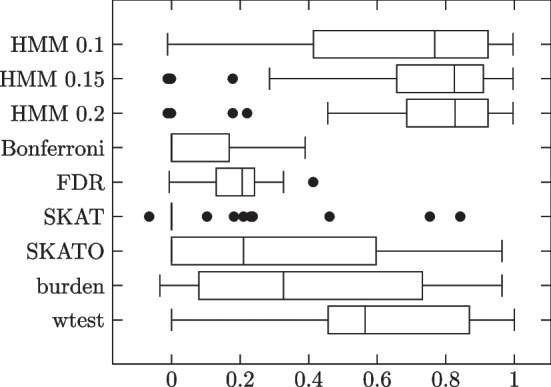
Individual trial MCCs

Lastly, we generated two groups of block model simulations with small sample sizes. In the first group, we used 150 phenotype 0 subjects and 150 phenotype 1 subjects. In the second group, we used 200 phenotype 0 subjects and 100 phenotype 1 subjects. Due to the large variance in performance, the number of trials in each group was doubled to 40. Settings were as in the first group of block model simulations, except with uniform i.i.d. individual site strengths. Results summarized in Tables 8, 9 and Figs. 9, 10.

**Table 8:** Block model simulation results: 150 phenotype 0, 150 phenotype 1 subjects

Method	Sensitivity	Specificity	MCC
HMM 0.1	0.8220	0.9483	0.7605 (0.2191)
HMM 0.15	0.8202	0.9843	0.8300 (0.1299)
HMM 0.2	0.8176	0.9893	0.8429 (0.1250)
Bonferroni	0.0164	1.000	0.0782 (0.0984)
FDR	0.1232	0.9884	0.1964 (0.0786)
SKAT	0.1099	0.9792	0.0948 (0.2103)
SKATO	0.2921	0.9792	0.3324 (0.3060)
Burden	0.3279	0.9605	0.3289 (0.2956)
wtest	0.6496	0.9695	0.6577 (0.2653)

Influential site strengths independent of MAFs. No added noise terms. Pure blocks

**Table 9:** Block model simulation results: 200 phenotype 0, 100 phenotype 1 subjects

Method	Sensitivity	Specificity	MCC
HMM 0.1	0.6765	0.9545	0.6279 (0.3620)
HMM 0.15	0.7257	0.9868	0.7178 (0.2885)
HMM 0.2	0.7371	0.9896	0.7398 (0.2716)
Bonferroni	0.0196	1.000	0.0831 (0.1037)
FDR	0.1609	0.9843	0.1903 (0.0846)
SKAT	0.0883	0.9884	0.0798 (0.1983)
SKATO	0.3554	0.9741	0.3370 (0.3386)
Burden	0.3940	0.9670	0.3926 (0.3317)
wtest	0.6602	0.9540	0.6022 (0.2458)

Influential site strengths independent of MAFs. No added noise terms. Pure blocks

While all three HMM thresholds outperform the comparison methods, there is a clear preference for higher threshold values, with HMM 0.2 doing best.

### Block model with low MAFs

**Fig. 11:**
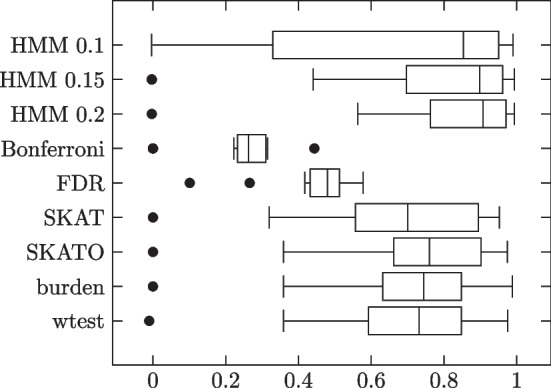
Individual trial MCCs

Lastly, bearing in mind that ZFA was primarily designed for testing rare variants, we generated a group of block model simulations with low MAFs. MAFs for each site were set i.i.d. to $q=0.001\cdot 10^u$, where $u\sim U(0,1)$. That is, MAFs could range from $0.1\%$ to $1\%$. To compensate for the low MAFs, i.e. to capture a sufficient number of subjects with the minor alleles, sample size was increased to 2500 phenotype 0 subjects and 2500 phenotype 1 subjects. Other settings were as in the first group of block model simulations, except with uniform i.i.d. individual site strengths. Results summarized in Table 10 and Fig. 11.

**Table 10:** Block model simulation results: 2500 phenotype 0, 2500 phenotype 1 subjects

Method	Sensitivity	Specificity	MCC
HMM 0.1	0.6706	0.9907	0.6402 (0.3950)
HMM 0.15	0.7492	0.9957	0.7510 (0.3184)
HMM 0.2	0.8117	0.9958	0.8139 (0.2441)
Bonferroni	0.0789	1.0000	0.2496 (0.1079)
FDR	0.4308	0.9831	0.4545 (0.1158)
SKAT	0.8103	0.9716	0.6705 (0.2533)
SKATO	0.7347	0.9899	0.7171 (0.2436)
Burden	0.6946	0.9920	0.6996 (0.2455)
wtest	0.7099	0.9902	0.6933 (0.2460)

Influential site strengths independent of MAFs. Low MAFs. No added noise terms. Pure blocks

We again see a preference for higher HMM threshold values. HMM 0.15 outperformed the comparison methods in mean MCC, but at the cost of higher variance. HMM 0.2 achieved the highest mean MCC of them all, with lower variance than the ZFA methods.

## Discussion

### Identification of significant genes

HMM is only designed to detect influential sites, not genes directly. The latter is generally the next step in studying genetic causal pathways for the phenotype [28]. We suggest the simplest approach: If a site is marked as influential, look for a gene on which it may exert a direct influence, i.e. a gene for which the site is either in the coding region or the regulatory region [13].

### Comparison with other methods

Both HMM and ZFA improve on individual site testing methods by using information from neighboring sites, on the presumption that influential sites come in clusters. However, HMM has several advantages. It offers greater versatility because it is designed for both rare and common variants, whereas ZFA is designed for just rare variants and wtest for common variants. Furthermore, ZFA predicts that influential sites come in (pure) blocks. This may be too idealized an assumption. HMM offers more flexibility because while it prefers purity, by disfavoring too many transitions to different states, it does not insist on it.

### Directions for future improvements

We suggest four possible modifications to our method.

First, the method assumes that all sites in the same state exert the same influence strength on the phenotype, as summarized by two parameters ($\theta _0^-$ and $\theta _1^-$ for Negative Influence sites, $\theta _0^+$ and $\theta _1^+$ for Positive Influence sites). But this assumption is simplistic; we should expect that in reality, some influential sites will exert a stronger influence than others. An alternative would be for individual Negative Influence (respectively Positive Influence) sites to have their strengths drawn i.i.d. from distributions. The $\theta$ parameters would then be interpreted not as the strengths of individual sites, but as the means of these distributions. We experimented with such a *hierarchical* HMM model, using exponential distributions for site strengths; but found that the runtime was unacceptably high. However, with algorithmic improvements or more computational resources, a hierarchical HMM model could be a direction for future research.

Secondly, the method assumes that the transition probability from state *k* to state $\ell$ should a priori be constant. But this assumption too is simplistic. In a real GWAS, the variant sites studied will not be equally spaced; some variant sites will be much closer to their neighboring sites than others. We should expect greater preference for remaining in the same state, i.e. larger diagonal values of the Markov transition matrix, when a site is close to its successor. The simplest way to model for this would be to insert a *breakpoint* whenever the number of base pairs between a site and its successor is larger than some threshold. Then the genome would be divided into segments bounded by these break points, and HMM run separately on each segment. After HMM has finished on all the segments, we could either accept the outputs as is or pool the parameter estimates for $\theta ,\pi ,A$ across all the segments and use those to re-estimate the most probable state sequence in each segment one last time. The advantage of this approach is that it splits most of the computation into separate jobs that can be run in parallel. But a disadvantage is that it still treats all transitions from state *k* to state $\ell$ in a single segment as a priori the same. Furthermore, if the GWAS contains a cluster of a few, or even just one, variant site far away from all other sites, this will result in a very short segment, on which it is not possible to make good inferences for Markov transition probabilities.

An alternative is to make the transition probabilities functions of genomic distance between a site and its successor. Small distances would yield “distinctive” transition probabilities with large diagonal values, while large distances would in the limit converge towards a background probability distribution $\pi$, as if the next site were a fresh start.

Thirdly, Markov chains are memoryless, in the sense that after just one step, the state at the current step becomes irrelevant to subsequent steps, a property that HMM inherits. This is not the best fit for accurately classifying variant sites in influential blocks with impurities because the correct classification of even a single No Influence site renders irrelevant all previous correct classifications of Negative/Positive Influence sites to classifications of later sites. It would be preferable for the model to “remember” nearby influential sites so that it will be more likely to correctly classify influential sites while still within an impure influential block. For this, we may look to *k*th order Markov chains, where the state of a site depends on the states of *k* preceding sites, and from there develop an analogous theory of *k*th order HMMs. We caution however that the size of the transition matrix grows as $3^k$, and with it the computational cost.

Fourthly, an even more interesting direction for future work would be to develop a bi-directional version of our method; a theory of bi-directional HMMs is introduced in [33].

## Conclusions

In this paper, we developed a Hidden Markov Model for the classification of influential sites using data from a GWAS. Our model assumes that states come in three states: Negative Influence, No Influence and Positive Influence. Each state has an emission distribution function, which assigns probabilities to the contingency tables at each variant site. The No Influence state assumes that phenotype 1 subjects have the same distribution of genotypes as phenotype 0 subjects, while the Negative Influence and Positive Influence states are departures from this null, with magnitude of departure controlled by two parameters each. The states themselves are governed by a Markov process, with a starting state probability vector for the first site and a transition probability matrix when passing from one site to the next. Our algorithm accepts as input a matrix of genotypes and a vector of phenotypes from a GWAS, makes some initial estimates for the model parameters, and alternates between updating the most probable state sequence and updating the model parameters, until finally halting and outputting its best estimate of the most probable state sequence.

Our model provides a mechanism for why influential sites should tend to cluster into blocks: Each site affects its successor because staying in the same state is a priori more probable than transiting to a different state. The model offers versatility, in being designed for both rare and common variants, and flexibility, in preferring influential sites to come in uninterrupted blocks but without insisting on it. Across diverse groups of simulations based on block models of influential sites, HMM consistently outperforms both simple comparison methods (Fisher’s exact test with corrections to the *p* value significance threshold) and more complex comparison methods (ZFA with four different algorithms to obtain subsequence *p* values). We anticipate that HMM may offer improved performance in classifying influential sites from GWAS, a strong first step in the study of genotype-phenotype causal relationships.

## Figures and Tables

**Fig. 12:**
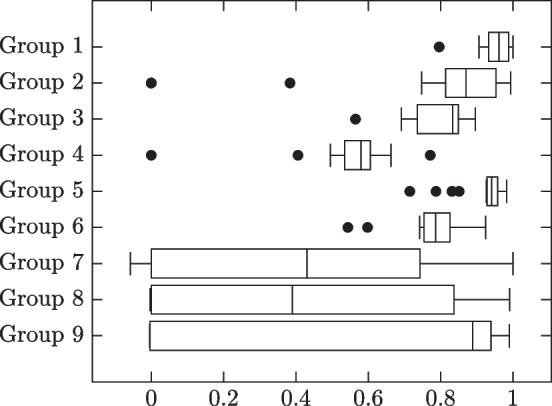
Individual trial MCCs

## Data Availability

The datasets used and/or analysed during the current study are available from the corresponding author on reasonable request.

## Appendix

### Explanation of emission distribution functions

Recall that in the No Influence State, $q_i=p_i$, whereas in the Negative Influence and Positive Influence states, the $q_i$ depart from $p_i$ according to the formulas in Emission Distribution Functions. This section will explain the reason for those formulas. Begin by noting that under Negative Influence, we expect more phenotype 1 subjects to have the AA genotype and less to have the aa genotype, while under Positive Influence, we expect less phenotype 1 subjects to have the AA genotype and more to have the aa genotype. However, such departures should avoid extreme changes to the MAF; rare alleles should not become common, nor common alleles become rare. We accomplish this using the softmax transformation.

Let $\varphi (t_0,t_1,t_2)=(\frac{e^{t_0}}{e^{t_0}+e^{t_1}+e^{t_2}},\frac{e^{t_1}}{e^{t_0}+e^{t_1}+e^{t_2}},\frac{e^{t_2}}{e^{t_0}+e^{t_1}+e^{t_2}})$. This is a transformation that takes triples of real numbers to triples of positive numbers that sum to 1. It has the following properties:

- If $\varphi (t_0,t_1,t_2)=(p_0,p_1,p_2)$, then one possible choice is $t_i=\log (p_i)$. However, it is not the only choice because of the following
- $\varphi (t_0,t_1,t_2)=\varphi (t_0+c,t_1+c,t_2+c)$ for any *c* This means for a given triple of probabilities $(p_0,p_1,p_2)$, the triple that maps to it is well-defined only up to adding a constant to all entries.
- If $\varphi (t_0,t_1,t_2)=(p_0,p_1,p_2)$ and $\varphi (t_0+c,t_1,t_2)=(p_0',p_1',p_2')$ with $c>0$, then $p_0'>p_0$, $p_1'<p_1$ and $p_2'<p_2$. Similarly for both other positions This means that increasing one entry transfers mass from the other two probabilities to the corresponding probability.

Specifically, we take $t_i=\log (p_i)$, shift the $t_i$, and then transform back using $\varphi$. For Negative Influence, we use the shifts $t_0=t_0+\theta ^-_0$ and $t_2=t_2-\theta ^-_1$. For Positive Influence, we use the shifts $t_0=t_0-\theta ^+_0$ and $t_2=t_2+\theta ^+_1$. This yields the formulas for $q_i$ in Emission Distribution Functions.

### M step of EM algorithm

In this section, we will need to maximize functions in which all arguments must be positive, and triples of arguments must sum to 1. That is, functions of the form $f(x_0,x_1,\ldots ,x_{3k+2})$ where $x_0,\ldots ,x_{3k+2}>0$ and $x_{3i}+x_{3i+1}+x_{3i+2}=1$ for $i=0,\ldots ,k$.

Consider first the simplest case, where the domain of *f* consists of only one such triple. We will use a transformation $h:\{(x_0,x_1,x_2):x_0,x_1,x_2>0,x_0+x_1+x_2=1\}\rightarrow [0,1]^2$ with $h(x_0,x_1,x_2)=(t,u)$, where $t=x_0$ and $u=\frac{x_1}{x_1+x_2}$. The idea is to transform the problem of maximizing a function on $\{(x_0,x_1,x_2):x_0,x_1,x_2>0,x_0+x_1+x_2=1\}$ to maximizing on $[0,1]^2$ via *h*. Next, we maximize with respect to *t* while fixing *u*, then maximize with respect to *u* while fixing *t*. Finally, we transform back using $h^{-1}(t,u)=(t,u(1-t),(1-u)(1-t))$.

More generally, the procedure we use for maximizing these functions is as follows:

1. Initialize starting values of $x_0,\ldots ,x_{3k+2}$
2. For $$i=0,\ldots ,k$$:
  - Regard *f* as a function of only $x_{3i},x_{3i+1},x_{3i+2}$, with all other variable values temporarily fixed.
  - Apply the procedure of the previous paragraph. Update values of $x_{3i},x_{3i+1},x_{3i+2}$
3. Repeat Step 2 until the value of $f(x_0,\ldots ,x_{3k+2})$ increases by $\le 10^{-8}$

Call this procedure triplet optimization.

Turning to the main problem of this section, recall the function we wish to maximize: $\sum _{j=1}^p\sum _{s\in \{-1,0,1\}}\gamma _j^{(t)}(s)(\log (f_s^j(x_j| \vec{p} ,\theta ))+\log P(S_j=s|\pi ,A,S_{j-1}))$, over the parameters $\vec{p} ,\theta ,\pi ,A$. This problem naturally splits into two components:

- Maximize $$\sum _{j=1}^p\sum _{s\in \{-1,0,1\}}\gamma _j^{(t)}(s)\log (f_s^j(x_j| \vec{p} ,\theta ))$$ over $$\vec{p} ,\theta$$
- Maximize $$\sum _{j=1}^p\sum _{s\in \{-1,0,1\}}\gamma _j^{(t)}(s)\log P(S_j=s|\pi ,A,S_{j-1})$$ over $$\pi ,A$$

#### Maximization over $\vec{p} ,\theta$

This part calls for maximizing a function with 3*p* terms over $2p+4$ independent variables, which is computationally too costly. To simplify, we maximize over these sets of variables one at a time. More specifically, our algorithm works as follows:

1. Initialize $\vec{p} ,\theta$ to their current values $\vec{p} ^{(t)},\theta ^{(t)}$
2. Maximize with respect to $\vec{p}$ while holding $\theta$ constant and update values of $\vec{p}$
3. Maximize with respect to $\theta$ while holding $\vec{p}$ constant and update values of $\theta$
4. Repeat Steps 2 and 3 until objective function increases by less than $10^{-8}$

In step 2, since $[p_0^j,p_1^j,p_2^j]$ is now free to vary independently for each variant site *j*, we maximize $\sum _{s\in \{-1,0,1\}}\gamma _j^{(t)}(s)\log (f_s^j(x_j| \vec{p} ,\theta ))$ independently for each *j* using triplet optimization.

For step 3, note that terms with $s=-1$ depend on only $\theta ^-_0,\theta ^-_1$ while terms with $s=1$ depend on only $\theta ^+_0,\theta ^+_1$; and terms with $s=0$ do not depend on $\theta$ at all. So this step reduces to

- Maximize $\sum _{j=1}^p\gamma _j^{(t)}(-1)\log (f_{-1}^j(x_j| \vec{p} ,\theta ))$ with respect to $\theta ^-_0,\theta ^-_1$
- Maximize $\sum _{j=1}^p\gamma _j^{(t)}(1)\log (f_1^j(x_j| \vec{p} ,\theta ))$ with respect to $\theta ^+_0,\theta ^+_1$

Each of these is a two-variable optimization problem. We solve them by maximizing with respect to the first variable while holding the second constant, then maximizing with respect to the second variable while holding the first constant. These single-variable searches are done over the interval [0, 10]. This loop is repeated until the objective function improves by $\le 10^{-8}$.

#### Maximization over $\pi ,A$

We use the classical Baum–Welch formulas:

$$\begin{aligned} \pi ^{(t+1)}&= \gamma _0^{(t)},\\ A_{k\ell }^{(t+1)}&= \frac{\Sigma _{j=1}^{p-1}\zeta _j^{(t)}(k,\ell )}{\Sigma _{j=1}^p\gamma _j^{(t)}(k)}, \end{aligned}$$

where $\zeta _j(k,\ell )$ is the marginal probability of being in state *k* at site *j* and state $\ell$ at site $j+1$; it too is explained in [30].

### Threshold sensitivity analysis

A question researchers using our method should consider is the choice of values for the thresholding parameters $\theta _{min}$ and $a_{min}$. Higher values mean stronger prior assumptions. Therefore, from a conceptual point of view, lower values are preferable. A Markov diagonal threshold value of $a_{min}=0.5$ assumes that sites in any state should be at least as likely to stay in the same state as to change states. This is already a conservative assumption, and given HMM’s consistently better performance than the comparisons, it is adequate. Therefore, we can confidently recommend using $a_{min}=0.5$.

More challenging is the choice of value for $\theta _{min}$. Too small a value, and HMM may expect little difference between influential and non-influential sites, causing a loss of sensitivity or specificity. Indeed, in our experience, a threshold value of 0.05 sometimes produced worse results than the comparison methods. Table 11 and Fig. 12 summarize the performance of HMM 0.05 in each of the simulation groups, using the same performance measures.

**Table 11:** Performance of HMM 0.05 in all simulation groups

Group	Sensitivity	Specificity	MCC
1	0.9945	0.9930	0.9512 (0.0486)
2	0.7775	0.9879	0.7625 (0.3110)
3	0.8587	0.9696	0.7928 (0.0846)
4	0.6296	0.9597	0.5464 (0.1556)
5	0.9115	0.9943	0.9161 (0.0699)
6	0.8804	0.9702	0.7768 (0.0859)
7	0.7406	0.6377	0.3817 (0.3859)
8	0.7130	0.6949	0.4077 (0.3923)
9	0.6451	0.9957	0.6288 (0.4294)

Groups are numbered in the order they are listed in the main paper

On the other hand, too large a value will force HMM to expect a stronger signal from influential sites than is actually the case. Based on our simulation results, we generally expect that any choice within the range [0.1, 0.2] should be ok, with a small preference for 0.15 for being in the middle of this tested range. However, some of the groups showed significant differences in performance depending on $\theta _{min}$. In the Small Sample Size groups, higher threshold values are preferred. We expect this is due to the behavior of the multinomial distribution; the smaller the sample size, the larger the relative width of the distribution, and hence the smaller the differences between the emission distribution functions of different states. A larger $\theta$ threshold is thus needed to compensate. Larger threshold values are also preferred for the low MAFs group. However, the first block model simulation group shows just the opposite trend; lower threshold values are preferred. The distinctive feature of this group is the dependence of individual site strength on MAF. Perhaps a lower threshold is needed to correctly identify influential sites with common minor alleles with weak influence.
